# Systemische Barrieren verhindern eine kompetenzbasierte Lehre im Bereich Planetary Health: Eine studentische Online-Befragung und deskriptive Analyse des Nationalen Kompetenzbasierten Lernzielkatalog Medizin 2.0

**DOI:** 10.1007/s00101-025-01525-6

**Published:** 2025-03-22

**Authors:** Philippe Kruse, Mark Coburn, Florian Windler, Birgit Bette, Christian Bode, Se-Chan Kim, Achilles Delis, Maria Wittmann, Gregor Massoth

**Affiliations:** 1 Abteilung für Anästhesiologie und Operative Intensivmedizin, Universitätsklinikum Bonn, Bonn, Deutschland; 2 Universitätsklinikum Bonn, Venusberg-Campus 1, 53127 Bonn, Deutschland; 3 Department of Anesthesiology, Montefiore Medical Center, Bronx, New York, USA

**Keywords:** Klimabedingte Gesundheitsfolgen, Studierende, Curriculum, Nachhaltigkeit, Umfrage, Climate-related health impacts, Students, Curriculum, Sustainability, Survey

## Abstract

**Hintergrund:**

Die aktuelle medizinische Studierendenschaft gehört zu der Ärztegeneration, welche die gesundheitlichen Folgen des Klimawandels bei der ärztlichen Tätigkeit bemerken wird. Dieses Wechselspiel zwischen der Integrität der Ökosysteme und der menschlichen Gesundheit untersucht die multidisziplinäre Forschungsdisziplin Planetary Health. Zur Bewältigung der klimabedingten Gesundheitsfolgen muss den Studierenden ein fundiertes Wissen von den Folgen zerstörter Ökosysteme auf die menschliche Gesundheit vermittelt werden. Trotz der Aktualität dieser Thematik zeigen aktuelle Studien ein unzureichendes Lehrangebot in diesem Bereich. In dieser Studie sollen systemische Barrieren, die eine kompetenzbasierte Lehre im Bereich Planetary Health verhindern, identifiziert werden.

**Methode:**

Die Analyse systemischer Barrieren erfolgte zuerst mittels einer anonymen Online-Befragung von Studierenden, welche das verpflichtende Blockpraktikum „Anästhesiologie“ im Wintersemester 2021/2022 belegten hatten. Als systemische Barriere wurden Hindernisse, welche eine kompetenzbasierte Lehre im Bereich Planetary Health behindern, definiert. Weiterführend wurde die Häufigkeit von definierten Begriffen, welche im Zusammenhang mit Planetary Health stehen, bestimmt. Dieses diente zur Analyse der Integration von Planetary Health in den „Nationalen Kompetenzbasierten Lernzielkatalogs Medizin 2.0“ (NKLM 2.0).

**Ergebnisse:**

Von 130 Studierenden, die das Blockpraktikum im Wintersemester 2021/2022 belegten, gaben 54 % der Studierenden (*n* = 70) einen vollständig ausgefüllten Fragebogen ab. Der Aussage, dass sich der Klimawandel negativ auf die Gesundheit der Patient:innen auswirken wird, stimmten 96 % (*n* = 67) der Studierenden zu. Bei 53 % (*n* = 37) der Teilnehmenden war Planetary Health bisher kein Bestandteil einer Lehrveranstaltung gewesen. Gleichfalls finden Begriffe, die im thematischen Zusammenhang mit Planetary Health stehen, nur selten Verwendung im NKLM 2.0.

**Schlussfolgerung:**

Zwei systemische Barrieren für eine kompetenzbasierten Lehre im Fach Planetary Health wurden identifiziert. Zum einem sind zu wenige verpflichtende Lehrveranstaltungen als Zeichen einer unzureichenden Verankerung in das Curriculum zu nennen. Zum anderen ist eine nichtausreichende Integration von Planetary Health im NKLM 2.0 aufzuführen. Die aktuelle Reform des NKLM 2.0 stellt eine Chance zur Überwindung dieser Barrieren dar.

## Abstract (en)

**Background:**

The current medical studentship will be a generation of doctors who will experience the effects of climate change on global health during their clinical practice. This interplay between the integrity of ecosystems and human health is being investigated in the multidisciplinary research field planetary health. To cope with these climate-related health effects, students must be given a profound knowledge of the impact of destabilized natural systems on human health; however, despite the actuality of this topic recent studies show that competence-based teaching on this topic is insufficient. Considering the relevance of this topic, this study aim to identify systemic barriers preventing competence-based teaching in the field of planetary health.

**Methods:**

Systemic barriers were first analyzed by means of an anonymous online survey of students. Students who completed the compulsory internship in anesthesiology in the winter semester 2021/2022 were invited to take part in this survey. Obstacles preventing competence-based teaching in the field of planetary health were defined as systemic barriers. Secondly, the frequency of defined terms related to planetary health was determined. This analysis was carried out to investigate the integration of planetary health into the “National Competence-based Catalogue of Learning Objectives for Medicine 2.0” (NKLM 2.0).

**Results:**

Out of 130 students who participated in the internship, 54% (*n* = 70) submitted a completed questionnaire. Of these students 96% (*n* = 67) agreed with the statement that climate change will have a negative impact on patients’ health. For 53% (*n* = 37) of participants, planetary health had not previously been part of teaching courses. In accordance, terms that are thematically related to planetary health were rarely used in the NKLM 2.0.

**Conclusion:**

The study identified two systemic barriers to competence-based teaching in the subject of planetary health. The first barrier is the low number of compulsory classes which represents the insufficient integration of planetary health into the curriculum. Secondly, the subject of planetary health has not yet been sufficiently integrated into the NKLM 2.0. The current reform of the NKLM 2.0 represents an opportunity to overcome these two barriers and to implement competence-based teaching through joint efforts.

Im Jahr 2024 ist das im Pariser Klimaabkommen vereinbarte 1,5 °C-Ziel erstmalig überschritten worden. Infolge der anhaltenden menschengemachten Zerstörung natürlicher Lebensräume werden die klimabedingten Gesundheitsfolgen und Gesundheitsrisiken in den kommenden Jahren stark zunehmen. Für eine adäquate Gesundheitsprävention und medizinische Versorgung ist eine umfassende Ausbildung der Studierenden als zukünftige Ärzt:innen im Bereich Planetary Health unabdingbar. Für eine zeitnahe Implementierung einer kompetenzbasierten Lehre müssen systematische Barrieren identifiziert und abgebaut werden.

## Einführung

Infolge der zunehmenden Globalisierung und des technologischen Fortschritts ist der Mensch zur dominierenden Kraft von ökologischen Prozessen geworden („great acceleration“). Dabei hat die Menschheit bis heute mehrere planetare Grenzen überschritten. Mit Planetary Health entstand in der jüngeren Vergangenheit eine transdisziplinäre Gesundheitsdefinition, welche die Integrität der ökologischen Systemen als Gesundheitsdeterminante untersucht [17, 18].

Die steigende Inzidenz und die geografische Ausbreitung einer Vielzahl an Erkrankungen können auf klimabedingte Veränderungen zurückgeführt werden [9]. Die durch den Klimawandel gesteigerte Wahrscheinlichkeit von extremen Temperaturlagen, Unwettern und schweren Niederschlägen geht ebenfalls mit einer gesteigerten Mortalität und Morbidität einher [8, 10]. Des Weiteren behindern die ökologischen Folgen des Klimawandels den sicheren Zugang zu Nahrung und sauberem Wasser in einigen Regionen der Welt [27, 36]. Ebenfalls sind die Auswirkungen der klimatischen Veränderungen auf die mentale Gesundheit in Form von chronischem Stress und Existenzängsten nicht zu vernachlässigen [41]. Damit stellt der Klimawandel nicht nur ein schwer zu kalkulierendes Gesundheitsrisiko dar, sondern ist die größte medizinische Herausforderung für das 21. Jahrhundert [42]. Die Umsetzung von weitgreifenden Klimaschutzmaßnahmen wäre eine effektive Maßnahme zur Minimierung dieser Gesundheitsrisiken [37]. Eine Begrenzung des globalen Temperaturanstiegs auf 1,5 °C anstatt auf 3 °C würde in Deutschland 1000 Herzinfarkte/Jahr verhindern [6]. Gleichfalls könnte der weltweite Verzicht auf fossile Brennstoffe jährlich 3,6 Mio. vorzeitige Todesfälle verhindern [23].

In Anbetracht der anhaltenden Überschreitung der planetaren Grenzen und der damit verbundenen Gesundheitsfolgen ist eine kompetenzbasierte Lehre im Bereich Planetary Health von zunehmender Bedeutung [19, 28]. Im Zuge der Dringlichkeit haben internationale Organisationen, medizinische Fachgesellschaften und Universitäten Initiativen und Absichtserklärungen zur Integration von Planetary Health in das Curriculum verabschiedet [43]. Dennoch gaben in einer weltweiten Befragung der International Federation of Medical Students’ Associations (IFMSA) nur 15 % der medizinischen Hochschulen an, dass Planetary Health Bestandteil ihres Curriculums sei [28]. Diese Ergebnisse unterstreichen die Diskrepanz zwischen dem Bedarf an Lehre im Bereich Planetary Health und dem aktuellen Angebot, das häufig auf Wahlfachangebote beschränkt ist [11, 13].

In dieser Forschungsarbeit wurden durch eine Studierendenbefragung sowie eine Analyse des „Nationalen Kompetenzbasierten Lernkatalog 2.0“ (NKLM 2.0) systemische Barrieren eruiert, die eine kompetenzbasierte Lehre des Faches Planetary Health verhindern.

## Methodik

Bei der Analyse von systemischen Barrieren einer kompetenzbasierten Lehre im Bereich Planetary Health lag ein thematischer Schwerpunkt auf dem Teilbereich „Klimawandel und Nachhaltigkeit im Gesundheitswesen“. Hierzu wurden eine anonyme Online-Befragung der Studierenden und eine deskriptive Analyse des „Nationalen Kompetenzbasierten Lernzielkatalogs Medizin 2.0“ (NKLM 2.0) durchgeführt. Ein positives Ethikvotum (Lfd. Nr. 576/20) zur Durchführung der Online-Befragung wurde vorab eingeholt.

### Befragung zum Verantwortungsbewusstsein und zur Lehre von Planetary Health

Die Zielgruppe der anonymen Befragung waren die Studierenden der Humanmedizin an der Rheinischen Friedrich-Wilhelms-Universität, die das verpflichtende Blockpraktikum im Fach Anästhesiologie während des Wintersemesters 2021/2022 belegten. Der Teilnahmelink für den digitalen Fragebogen wurde den Studierenden bei der Einführungsveranstaltung des anästhesiologischen Blockpraktikums ausgehändigt. Eine Erinnerung erfolgte zu Beginn des jeweiligen Blockpraktikums. Der Befragungszeitraum erstreckte sich über das Wintersemester 2021/2022. Die Befragung und anschließende deskriptive Auswertung erfolgten mithilfe des webbasierten Umfrage-Tools SoSci Survey (SoSci Survey GmbH, München, Deutschland). Bei der Auswertung wurden nur vollständig ausgefüllte Fragebogen berücksichtigt.

Für die Identifikation potenzieller systemischer Barrieren untersuchte der Fragebogen (1) die studentische Sichtweise zu den erwarteten Konsequenzen des Klimawandels auf die öffentliche Gesundheit, (2) die persönliche und berufliche Verantwortung als angehender Ärzt:in in Bezug auf den Klimawandel und (3) die Integration von Lehrveranstaltungen mit dem Schwerpunkt Klimawandel in der ärztliche Ausbildung. Der Fragenkatalog wurde von den abteilungsinternen Nachhaltigkeitsbeauftragten (P.K., M.C., B.B., S.C.K., C.B., F.W.) sowie den Lehrverantwortlichen (A.D., M.W., G.M.) erarbeitet.

### Integration von Planetary Health in den nationalen Lernzielkatalog mittels einer deskriptiven Analyse

Für die Analyse der Integration von Planetary Health in den NKLM 2.0 wurde die aktuelle Version verwendet. Der NKLM 2.0 wird vom medizinischen Fakultätentag herausgegeben und dient der qualitativen Verbesserung der Lehre und des Studiums in der Humanmedizin. Bei der Analyse des NKLM 2.0 wurde nur der Hauptkatalog berücksichtigt, weil dieser als Kerncurriculum für die medizinischen Fakultäten verpflichtend ist. In seiner aktuellen Fassung besitzt der Hauptkatalog kein Kapitel zu Planetary Health. Daher wurde als Surrogatparameter für die Integration die Häufigkeit von Begriffen mit Bezug zu Planetary Health sowie von Klimaveränderungen mit nachgewiesener Gesundheitsgefahr analysiert. Die Analyse erfolgte mithilfe der plattformeigenen Suchfunktion. Zu den Begriffen gehörten (a) Klimawandel, (b) klimabedingt, (c) anthropogene Umweltveränderung, (d) Planetary Health und dessen deutsche Übersetzung sowie (e) planetare Gesundheit. Als Klimafolgen wurden (i) Hitzewelle, (ii) Luftverschmutzung und (iii) Naturkatastrophe untersucht. Anschließend wurde differenziert, ob die Begriffe im Zusammenhang mit Lernzielen oder als eingrenzende Erläuterungen genannt wurden. Wurden die Begriffe im Zusammenhang mit einem Lernziel genannt, wurde weitergehend nach dem Kompetenzniveau, das den Umfang des zu erwerbenden Wissens beschrieb, differenziert. Bei den Kompetenzniveaus wurde zwischen (I) Faktenwissen, (II) Handlungs- und Begründungswissen, (III) Handlungskompetenz unter Anleitung sowie (IV) selbstständiger Handlungskompetenz unterschieden.

## Ergebnisse

### Die Häufigkeit curricularer Lehrveranstaltungen zu Planetary Health wird von den Studierenden als zu gering wahrgenommen

Im Wintersemester 2021/2022 belegten 130 Studierende der Humanmedizin das verpflichtende Praktikum im Fach Anästhesiologie. An der Befragung nahmen 62 % der Studierenden (*n* = 81) teil, von denen 86 % (*n* = 70) einen vollständig ausgefüllten Fragebogen abgaben. Der Altersmedian der Teilnehmenden lag bei 22,5 Jahren (Tab. 1).

**Tab. 1:** Demografische Daten der Umfrageteilnehmenden (*n* = 70)

Frage	Antwort	Absolut (*n*)	Relativ (%)
*Verteilung der abgeschlossenen Fachsemester unter den Teilnehmenden*	7	6	9
	8	55	79
	9	3	4
	10	5	7
	11	1	1
*Nebentätigkeit im Gesundheitswesen*	Ja	35	50
	Nein	35	50
Ist bei der Nebentätigkeit ein Nachhaltigkeitskonzept vorhanden?	Ja	3	8
	Nein	15	43
	Ich weiß nicht	17	49
*Ehrenamt im Bereich Klimaschutz*	Ja	5	7
	Nein	65	93
Ist dieses Ehrenamt im Bereich des Gesundheitswesens?	Ja	1	20
	Nein	4	80

**Abb. 1:**
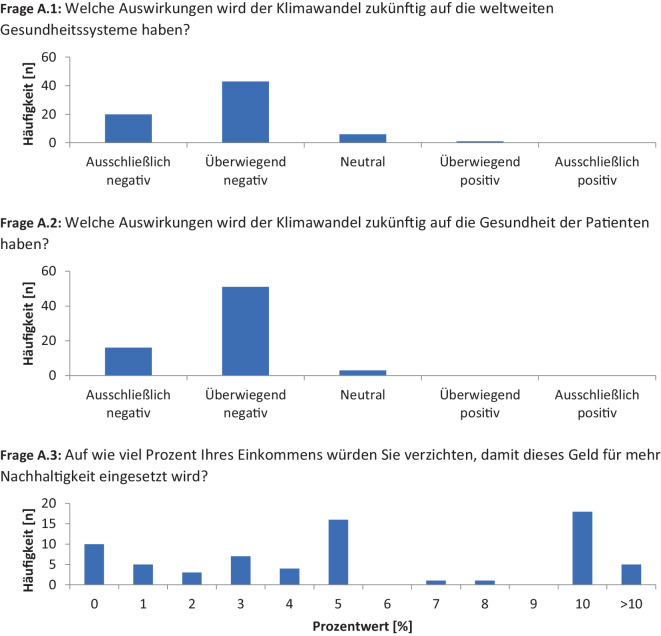
Fragenkatalog zur Analyse der studentischen Sichtweise zu den erwarteten Konsequenzen des Klimawandels auf die öffentliche Gesundheit und das persönliche Verantwortungsbewusstsein als systemische Barriere für eine kompetenzbasierte Lehre

Bei der Befragung widersprachen 10 % (*n* = 7) der Teilnehmenden der Aussage, dass der Klimawandel eine negative Auswirkung auf die weltweiten Gesundheitssysteme haben wird. Hingegen stimmten 90 % (*n* = 63) der Aussage zu (Abb. 1, Frage A.1). Des Weiteren gaben 96 % (*n* = 67) der Studierenden an, dass der Klimawandel negative Gesundheitsfolgen für die Patient:innen haben wird (Abb. 1, Frage A.2).

Das persönliche Verantwortungsbewusstsein der Studierenden in Bezug auf den Klimawandel wurde anhand eines potenziellen, monatlichen Gehaltsverzichts analysiert. Wenngleich 14 % (*n* = 10) der Teilnehmenden mit Gehaltseinbußen nicht einverstanden wären, würden 51 % (*n* = 36) der Befragten auf einen Betrag zwischen 5 und 10 % verzichten, wenn dieser für Nachhaltigkeitsmaßnahmen im medizinischen Sektor genutzt werden würde. Mit einem Gehaltsverzicht von mehr als 10 % waren 7 % (*n* = 5) der Studierenden einverstanden (Abb. 1, Frage A.3).

**Abb. 2:**
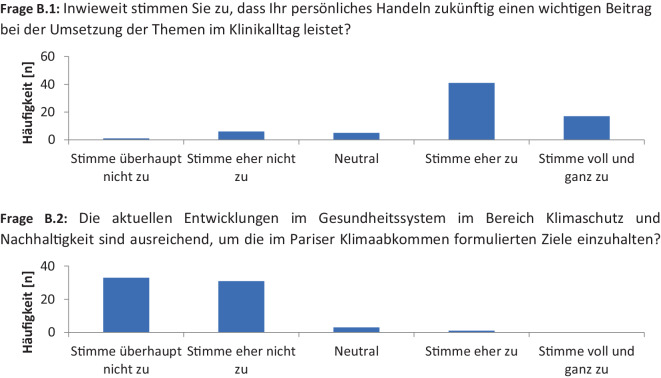
Fragenkatalog zur Analyse der beruflichen Verantwortung als angehender Ärzt:in in Bezug zum Klimawandel als systemische Barriere für eine kompetenzbasierte Lehre

Anschließend wurde das berufliche Verantwortungsbewusstsein untersucht. Von den Studierenden stimmten 83 % (*n* = 58) mit der Aussage überein, dass ihr ärztliches Handeln bei der Umsetzung von Nachhaltigkeitsprojekten von Relevanz sein würde (Abb. 2, Frage B.1). Die aktuellen Klimaschutz- und Nachhaltigkeitsmaßnahmen im Gesundheitssystem empfanden 94 % (*n* = 66) der Studierenden für unzureichend, um die im Pariser Klimaabkommen formulierten Ziele zu erreichen (Abb. 2, Frage B.2).

**Abb. 3:**
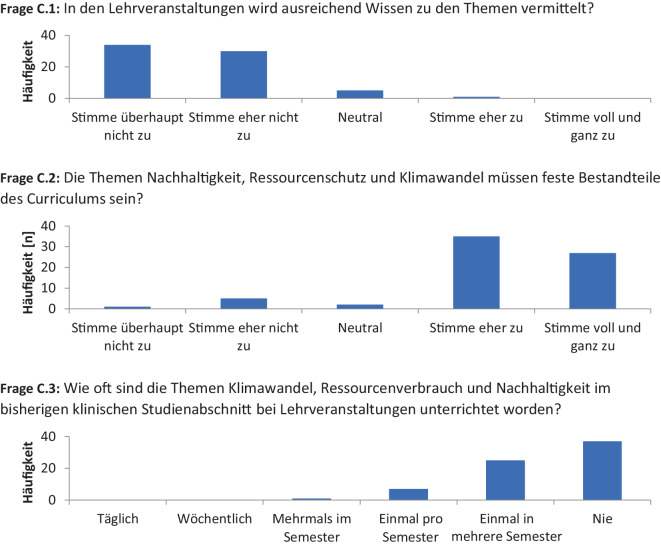
Fragenkatalog zur Analyse der Häufigkeit von Lehrveranstaltungen mit dem thematischen Schwerpunkt Klimawandel als systemische Barriere für eine kompetenzbasierte Lehre

Abschließend wurde die wahrgenommene curriculare Integration von Planetary Health untersucht. Das in den Lehrveranstaltungen vermittelte Wissen zum Thema Nachhaltigkeit und Klimaschutz im Gesundheitssystem empfanden 94 % (*n* = 66) der Teilnehmenden als unzureichend (Abb. 3, Frage C.1). Von den Teilnehmenden forderten 89 % (*n* = 62), dass die Themen Nachhaltigkeit und Klimaschutz Bestandteil des Curriculums sein sollten, wohingegen 9 % (*n* = 6) dieses verneinten (Abb. 3, Frage C.2). Die Häufigkeit von Lehrveranstaltungen, in denen die Themen Nachhaltigkeit und Klimaschutz adressiert wurden, gaben 12 % (*n* = 8) mit mindestens einmal pro Semester an. Bei 36 % (*n* = 25) der Teilnehmenden waren diese Themen weniger als einmal pro Semester thematisiert worden und bei 53 % (*n* = 37) in keiner der bisherigen Lehrveranstaltungen (Abb. 3, Frage C.3).

### Planetary Health wird in den Lernzielen des NKLM 2.0 nicht genannt

Der Begriff „Planetary Health“ wird im NKLM 2.0 nicht genannt, wobei die deutsche Übersetzung „planetare Gesundheit“ 2‑mal verwendet wird. Dabei wird der Begriff „planetare Gesundheit“ zur eingrenzenden Erläuterung der beiden Lernziele „Prinzipien pathogenetisch sowie salutogenetisch orientierter Therapien“ sowie „Begriffe, Modelle und Variablen von Public Health und Global Health benennen“ genannt. Der Begriff „Klimawandel“ wurde 26-mal genutzt und das Adjektiv „klimabedingt“ 5‑mal. Insgesamt 28-mal wurde der Begriff „anthropogene Umweltveränderung“ im NKLM 2.0 erwähnt. Von den analysierten Klimafolgen mit gesundheitlicher Relevanz wurden die Begriffe „Hitzewelle“ und „Luftverschmutzung“ 18-mal genannt. Hingegen wurde der Begriff „Naturkatastrophe“ nur einmal erwähnt. Keiner der Begriffe wurde im Kontext von Lernzielen verwendet, sondern lediglich zur eingrenzenden Erläuterung von diesen. Weil keiner der Begriffe im Zusammenhang mit Lernzielen genannt wurde, konnte keine Differenzierung nach den Kompetenzniveaus erfolgen.

## Diskussion

Die Ergebnisse dieser Studie zeigen, dass systemische Barrieren eine kompetenzbasierte Lehre im Bereich Planetary Health behindern. Für die aktuellen Studierenden ist eine fundierte Ausbildung von Bedeutung, weil diese als eine der ersten Ärztegenerationen die klimabedingten Gesundheitsfolgen in der vollen Ausprägung erleben werden [34]. Gleichzeitig haben Ärzt:innen als vertrauenswürdiger Berufsstand eine wichtige gesellschaftliche Vorbildfunktion mit weitreichender Ausstrahlkraft [35]. Ein Beispiel für eine erfolgreiche Gesundheitsaufklärung ist die Tabakprävention, die Jugendliche effektiv am Nikotinkonsum hinderte und die Exposition von Kindern gegenüber Zigarettenrauch minimierte [20].

Die teilnehmenden Studierenden waren mit den Grundprinzipien der Gesundheitsdefinition Planetary Health vertraut und stimmten den negativen Folgen der ökologischen Zerstörung für die Gesundheit zu. Internationale Studien konnten dieses ebenfalls zeigen [4, 14, 16, 29]. Damit ist ein unzureichendes Wissen über die klimabedingten Gesundheitsgefahren nicht als systemische Barriere für eine kompetenzbasierte Lehre anzusehen.

Bei dem Verantwortungsbewusstsein zeigte sich ein indifferentes Bild. Wir unterschieden zwischen dem persönlichen Verantwortungsbewusstsein, welches die Wahrnehmung von Aufgaben und Verpflichtungen in Bezug auf die Themen Nachhaltigkeit und Klimawandel im persönlichen Kontext beschreibt, und dem beruflichen Verantwortungsbewusstsein. Die persönliche Verantwortung der Studierenden reichte vom ehrenamtlichen Engagement bis hin zu einem Gehaltsverzicht für eine nachhaltige Transformation des Gesundheitssystems. Die Gleichstellung des persönlichen Verantwortungsbewusstseins mit einem Gehaltsverzicht basiert auf der Annahme, dass ein monetärer Verlust eine rationale Entscheidung ist. Wir konnten keine unterschiedliche Ausprägung zwischen dem persönlichen und beruflichen Verantwortungsbewusstsein feststellen. Hingegen zeigte eine Studie über Studierende, die sich im letzten Studienjahr befanden, ein schwächeres berufliches Verantwortungsbewusstsein [4]. Gleichfalls gaben Allgemeinmediziner:innen an, dass die Edukation von Patient:innen über den Zusammenhang zwischen Klimaschutz und Gesundheit nicht zu den ärztlichen Aufgaben gehört [2]. Damit stellt ein mangelndes berufliches Verantwortungsbewusstsein für die von uns befragten Studierenden keine systemische Barriere dar, jedoch sind aufgrund der nichteindeutigen Studienlage weitere Forschungsarbeiten notwendig.

Wenngleich die befragten Studierenden die aktuellen Transformationsbemühungen des Gesundheitssektors als unzureichend empfanden, hat das Thema in jüngster Vergangenheit in der klinischen Praxis an Bedeutung gewonnen. Zum einem haben verschiedene Fachgesellschaften Positionspapiere zur Etablierung von Nachhaltigkeits- und Klimaschutzmaßnahmen veröffentlicht [26, 31, 32]. Zum anderen untersuchen Studien die Effektivität von Maßnahmen für ein nachhaltiges Arbeiten im Gesundheitssektor [1, 3, 15, 30]. Gleichzeitig sahen die befragten Studierenden das bisherige Lehrangebot im Bereich Planetary Health als unzureichend an. Eine zu geringe Integration von Planetary Health in das Curriculum konnte auch in einer anderen Studie gezeigt werden [40]. Damit stellt eine unzureichende curriculare Integration in Form von zu wenigen Lehrveranstaltungen eine systemische Barriere dar. Hierbei ist als Limitation aufzuführen, dass die Dozierenden nicht Bestandteil der Befragung gewesen sind. Dementsprechend können keine Rückschlüsse auf die Ursache für das mangelnde Lehrangebot aus der Perspektive der Dozierenden gezogen werden. Als Möglichkeit könnte eine fehlende Awareness oder ein mangelndes Themenwissen in Betracht gezogen werden [25, 39]. Weitere Gründe könnten Prioritätenkonflikte infolge einer inhaltlichen Überladung des Curriculums oder eine fehlende interdisziplinäre Zusammenarbeit sein. Eine Ursache aufseiten der teilnehmenden Studierenden ist als unwahrscheinlich anzusehen, weil diese eine langfristige curriculare Implementierung von Nachhaltigkeit und Klimawandel wünschen. Folglich sollten die Ursachen des geringen Lehrangebots an den medizinischen Fakultäten durch weitere Studien systematisch erfasst und analysiert werden. Ein besonderes Augenmerk gilt dabei den Dozierenden.

Die deskriptive Analyse zur Integration von Planetary Health in die neue Fassung des NKLM 2.0 zeigte, dass sowohl Begriffe mit thematischem Bezug zu Planetary Health als auch Klimafolgen mit gesundheitlicher Relevanz mehrfach im NKLM 2.0 erwähnt werden. Jedoch erfolgte keine der Erwähnungen im Rahmen der Definition eines Lernzieles. Damit ist die unzureichende Integration von Planetary Health in den Lernzielkatalog des NKLM 2.0 als zweite systemische Barriere zu nennen. Diese Diskrepanz zwischen den Anforderungen an die studentische Ausbildung und der Adaptation des Curriculums wurde bereits in anderen Studien gezeigt [7, 12, 24]. Zwar ist die Veröffentlichung eines Zusatzkatalogs zu Planetary Health ein erster Schritt seitens des medizinischen Fakultätentags. Jedoch hat dieser nur einen fakultativen Charakter und fasst lediglich die Lernziele mit einem thematischen Bezug zu Planetary Health systematisch zusammen. Daher stellt der Zusatzkatalog keine Weiterentwicklung des NKLM im Hinblick auf Planetary Health dar.

Die Ergebnisse dieser Arbeit unterstreichen, dass systemische Barrieren bei der Implementierung einer kompetenzbasierten Lehre im Bereich Planetary Health existieren. Limitierend ist aufzuführen, dass bei diese Befragung durch den thematischen Schwerpunkt auf Hitzewellen, Luftverschmutzung und Naturkatastrophen nur ein Teilbereich der mannigfaltigen Disziplin Planetary Health abgebildet wurde [5, 22]. Wenngleich die teilnehmenden Studierenden ein durchschnittliches Semester widerspiegeln, handelt es sich hierbei um keine repräsentative Umfrage. Des Weiteren könnte die alleinige Berücksichtigung von vollständig ausgefüllten Fragebogen zu einer Verzerrung der Ergebnisse geführt haben, indem v. a. Studierende mit einem persönlichen Interesse den Fragebogen vollständig ausfüllten. Gleiches gilt für die Bereitschaft der Studierenden, an der Umfrage teilzunehmen.

Zusammenfassend lassen sich aus den Ergebnissen zwei wichtige systemische Barrieren für die Implementierung einer kompetenzbasierten Lehre ableiten, die es gilt, in weiterführenden Studien zu untersuchen. Nichtsdestotrotz wird bei der Integration von Planetary Health in den NKLM 2.0 und der Etablierung von Lehrveranstaltungen die Einbindung von Studierenden essenziell sein, um ein kompetenzbasiertes Lernkonzept auf der Basis ihrer spezifischen Bedürfnisse zu erstellen. Hierbei sollten Studierende und Lehrende als Teil eines kollaborativen Netzwerkes einbezogen werden [38]. „Meaningful student involvement“ ist ein bewährtes Konzept, das in der ärztlichen Ausbildung zunehmend an Bedeutung gewinnt. Dabei sollte die Analyse als spezielle Bedarfsanalyse nach Kern [21], Schritt 2 der Curriculumsplanung, erfolgen („targeted needs assessment“). Es ermöglicht, den Fokus der universitären Lehre von der reinen Wissensvermittlung hin zur Vermittlung von Kompetenzen und Fähigkeiten zu verlagern. Dieses befähigt die Studierenden, das Wissen in komplexe Zusammenhänge zu stellen, kritisch zu überprüfen und weiterentwickeln zu können [24]. Die Notwendigkeit, interdisziplinäre, präventive und globale Gesundheitsansätze in den Vordergrund zu stellen, und die bereits aufgeführte Einbindung der Dozierenden unterstreichen das Förderprogramm der American Medical Association [33].

## Fazit für die Praxis

- Die Studierenden sind sich der Konsequenzen durch den Klimawandel bewusst und besitzen sowohl ein persönliches als auch berufliches Verantwortungsbewusstsein. Jedoch spiegelt das aktuelle Lehrangebot zu Planetary Health die aktuellen medizinischen Anforderungen des Gesundheitssystems an die studentische Ausbildung nicht wider.
- Als systemische Barriere für eine kompetenzbasierte Lehre von Planetary Health identifizierten wir eine unzureichende Anzahl an Lehrveranstaltungen sowie eine nichtausreichende Integration von Planetary Health in den NKLM 2.0.
- Unsere Ergebnisse unterstreichen den Bedarf an ein erweitertes Lehrangebot im Bereich Planetary Health, um Studierenden ein hohes Kompetenzniveau in diesem Bereich zu vermitteln. Damit einhergehend sind die Ergebnisse eine Rationale für die Berücksichtigung von Planetary Health im Hauptkatalog des NKLM 2.0. Der aktuelle Reformprozess des NKLM stellt eine Möglichkeit dar, die entsprechenden Inhalte über den optionalen Zusatzkatalog hinaus zu integrieren.

## Data Availability

Die in dieser Studie erhobenen Datensätze können auf begründete Anfrage beim Korrespondenzautor angefordert werden.
